# A Transcriptomic-Phylogenomic Analysis of the Evolutionary Relationships of Flatworms

**DOI:** 10.1016/j.cub.2015.03.034

**Published:** 2015-05-18

**Authors:** Bernhard Egger, François Lapraz, Bartłomiej Tomiczek, Steven Müller, Christophe Dessimoz, Johannes Girstmair, Nives Škunca, Kate A. Rawlinson, Christopher B. Cameron, Elena Beli, M. Antonio Todaro, Mehrez Gammoudi, Carolina Noreña, Maximilian J. Telford

**Affiliations:** 1 Department Genetics, Evolution and Environment, University College London, Gower Street, London WC1E 6BT, UK; 2 Institute of Zoology, University of Innsbruck, Technikerstrasse 25, 6020 Innsbruck, Austria; 3 Department of Computer Science, University College London, Gower Street, London WC1E 6BT, UK; 4 ETH Zurich, Department of Computer Science, Universitätsstrasse 19, 8092 Zurich, Switzerland; 5 Université de Montréal, Département de Sciences Biologiques, Pavillon Marie-Victorin, CP 6128, Succ. Centre-ville, Montréal, QC H3C 3J7, Canada; 6 Dipartimento di Scienze e Tecnologie Biologiche ed Ambientali, Università del Salento, 73100 Lecce, Italy; 7 Università degli Studi di Modena e Reggio Emilia, Via Campi 213/d, 41100 Modena, Italy; 8 Université Tunis El-Manar Campus Universitaire, 2092 Tunis, Tunisia; 9 Museo Nacional de Ciencias Naturales (CSIC), José Gutiérrez Abascal 2, 28006 Madrid, Spain

## Abstract

The interrelationships of the flatworms (phylum Platyhelminthes) are poorly resolved despite decades of morphological and molecular phylogenetic studies [1, 2]. The earliest-branching clades (Catenulida, Macrostomorpha, and Polycladida) share spiral cleavage and entolecithal eggs with other lophotrochozoans. Lecithoepitheliata have primitive spiral cleavage but derived ectolecithal eggs. Other orders (Rhabdocoela, Proseriata, Tricladida and relatives, and Bothrioplanida) all have derived ectolecithal eggs but have uncertain affinities to one another. The orders of parasitic Neodermata emerge from an uncertain position from within these ectolecithal classes. To tackle these problems, we have sequenced transcriptomes from 18 flatworms and 5 other metazoan groups. The addition of published data produces an alignment of >107,000 amino acids with less than 28% missing data from 27 flatworm taxa in 11 orders covering all major clades. Our phylogenetic analyses show that Platyhelminthes consist of the two clades Catenulida and Rhabditophora. Within Rhabditophora, we show the earliest-emerging branch is Macrostomorpha, not Polycladida. We show Lecithoepitheliata are not members of Neoophora but are sister group of Polycladida, implying independent origins of the ectolecithal eggs found in Lecithoepitheliata and Neoophora. We resolve Rhabdocoela as the most basally branching euneoophoran taxon. Tricladida, Bothrioplanida, and Neodermata constitute a group that appears to have lost both spiral cleavage and centrosomes. We identify Bothrioplanida as the long-sought closest free-living sister group of the parasitic Neodermata. Among parasitic orders, we show that Cestoda are closer to Trematoda than to Monogenea, rejecting the concept of the Cercomeromorpha. Our results have important implications for understanding the evolution of this major phylum.

## Highlights

• Phylogenomics provide insights into the interrelationships of Platyhelminthes
• Macrostomorpha are the basalmost rhabditophorans
• Polycladida are sister group of Lecithoepitheliata/Prorhynchida
• Bothrioplanida are the free-living sister group of Neodermata

## Teaser

The interrelationships of the flatworms (phylum Platyhelminthes) are poorly resolved. Egger et al. assembled a phylogenomic dataset of >107,000 aligned amino acids with less than 28% missing data from 27 flatworm taxa in 11 orders covering all major clades and reconstruct a well-resolved tree with high confidence, revealing several unexpected clades.

## Results and Discussion

**Figure 1:**
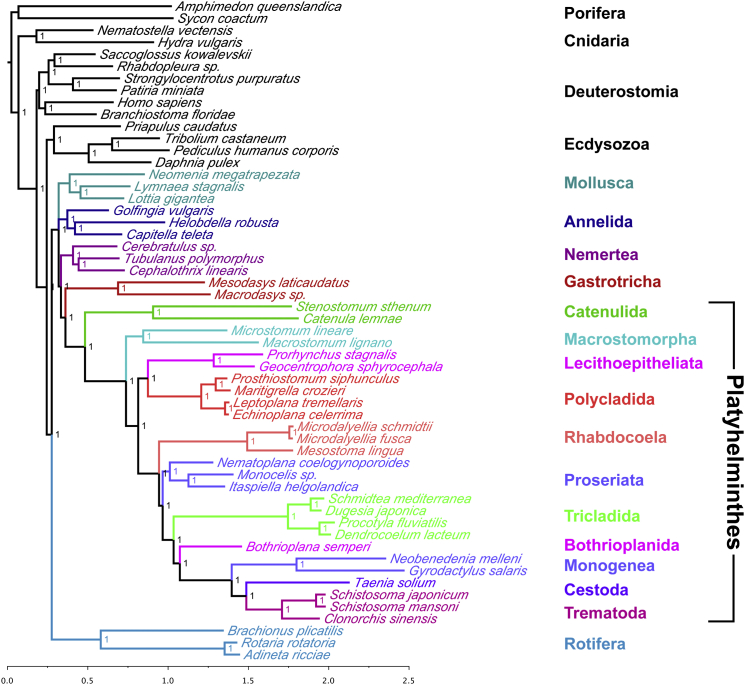
Phylogeny Produced Using PhyloBayes with the Site-Heterogeneous CAT+GTR+G4 Model on the Full 107,659 Amino Acid Alignment There is support for a sister group relationship between Gastrotricha and Platyhelminthes, which are members of an unresolved clade including mollusks, annelids, and nemerteans, contrary to the concept of the Platyzoa. Platyhelminthes are monophyletic. Macrostomorpha is the earliest-branching rhabditophoran clade. Lecithopepitheliata and Polycladida are sister groups. Rhabdocoels are the sister clade to all other neoophoran orders, including proseriates, but are separated from other Euneoophora by a very short internode. *Bothrioplana* is the closest free-living relative of the parasitic Neodermata. Values at nodes indicate posterior probabilities. Scale bar indicates number of substitutions per site. MaxDiff = 1.0; MeanDiff = 0.00934579. Lophotrochozoan groups in Figures 1, 2, and 3 are indicated by colored labels.

**Figure 2:**
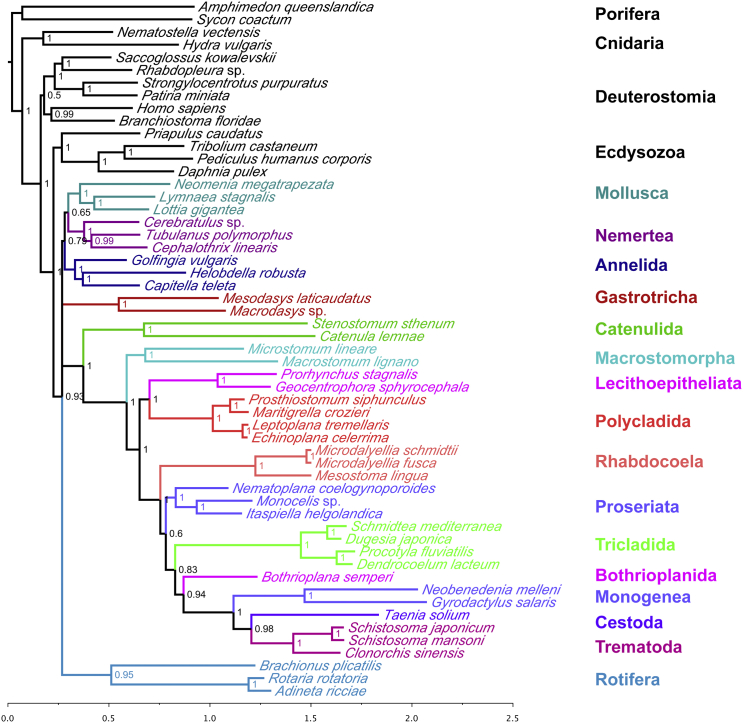
Jackknife Analysis of 100 Datasets of 20,000 Amino Acids Each, Produced Using the PhyloBayes CAT+GTR+G4 Model Values at nodes indicate proportion of replicates in which the node is found (1 corresponds to 100% jackknife). The topology is largely the same as the full analysis shown in Figure 1, and most clades receive high support. Relatively low support for the sister group relationship of rhabdocoels and other euneoophorans is observed. There is no clear support for or against Platyzoa, indicated by the polytomy at the base of the Lophotrochozoa. Scale bar indicates number of substitutions per site.

**Figure 3:**
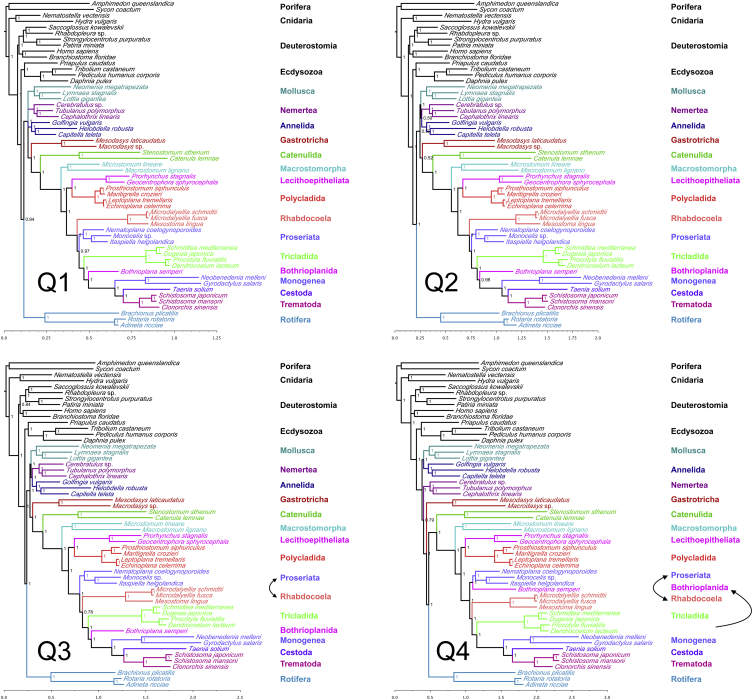
Phylogenetic Signal Dissection: Gene Rate Ranking to Look for Possible LBA Artifacts The trees shown were produced using PhyloBayes’ CAT+GTR+G4 model on four equal-sized datasets (quartiles Q1 to Q4) containing genes evolving at increasingly rapid rates (Figure S3). Q1 is slowest and expected to be least susceptible to long-branch attraction (LBA); Q4 is fastest evolving and, a priori, most susceptible to LBA. The trees of the slowest two quartiles are identical in all important respects to the topology found using the full dataset. In the faster-evolving quartiles, the positions of the long-branched rhabdocoels and short-branched proseriates are reversed. In Q4, the short-branched *Bothrioplana* groups with short-branched Proseriata and the long-branched Rhabdocoela and Neodermata are grouped together. In the slower-evolving Q1–Q3, no support for Platyzoa is observed. In Q4, support switches to Platyzoa (Rotifera, Gastrotricha, and Platyhelminthes), presumably due to LBA effects. Relative substitution rates: Q1 = 1.14, Q2 = 1.33, Q3 = 1.42, Q4 = 1.54. Percent missing data: Q1 = 27%, Q2 = 26%, Q3 = 28%, Q4 = 29%. MaxDiff/MeanDiff: Q1 = 1.0/0.0192412, Q2 = 0.928747/0.02347, Q3 = 0.425926/0.00683628, Q4 = 0.647856/0.00764029. Scale bars indicate number of substitutions per site.

We assembled coding sequence data from 55 animal species, including 27 species of platyhelminth. We identified 1,348 orthologous genes and produced a large (>107,000 positions) and taxonomically broad phylogenomic dataset (27 flatworm species from 11 orders) for the analysis of the phylogeny of this important and diverse group of animals. The dataset contains very few missing data (average 72% complete, measured as the percentage of positions with data present within the total alignment), especially in the case of the newly sequenced taxa (average 82% complete, all but two >68% complete). We used site-heterogeneous Bayesian tree reconstruction (PhyloBayes CAT+GTR+G4 [3] model, which has site-specific equilibrium frequency profiles; Figure 1) and site-homogenous maximum-likelihood (ML) approaches (PhyML LG+G4 [4] and RAxML CATGTR [5], which have homogenous equilibrium frequency profiles; Figures S1 and S2) to reconstruct the phylogeny based on these data. Most relationships within Platyhelminthes are robustly resolved as shown by concordance between different analyses, Bayesian posterior probabilities (Figure 1), jackknife resampling (Figure 2), and phylogenetic signal dissection (Figure 3).

### Platyhelminthes Are a Monophyletic Group of Lophotrochozoans

**Figure 4:**
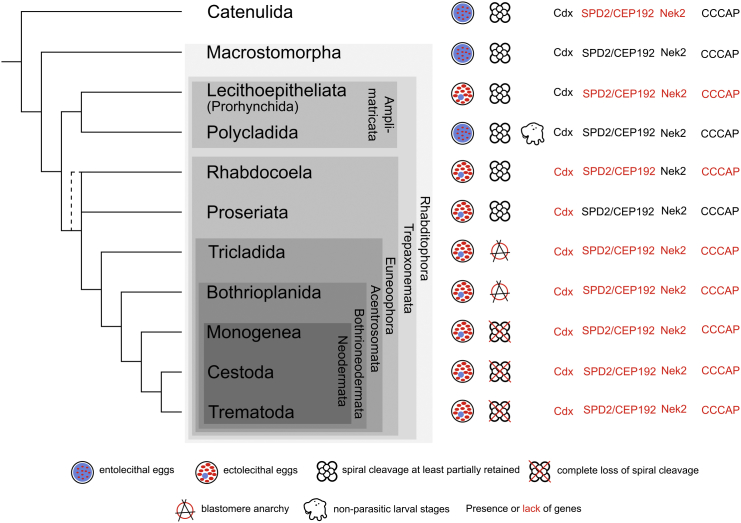
Consensus Tree of Relationships of Eleven Platyhelminth Orders with Important Morphological and Genetic Characters Mapped The less-reliably resolved branches involve the Rhabdocoela and the Proseriata, although our results suggest that basally branching Rhabdocoela (indicated by dashed line) is likely the correct solution. Developmental features, such as egg and cleavage type, planktotrophic larvae, and gene presence/absence patterns are indicated to the right of the tree. If polyclad larvae are homologous with the trochophores of annelids and mollusks, primary larval stages must have been lost in Catenulida, Macrostomorpha, Lecithoepitheliata, and the Euneoophora. Entolecithal eggs as found in Catenulida, Macrostomorpha, and Polycladida are an ancestral character. Ectolecithal eggs are independently present in Lecithoepitheliata and Euneoophora. The parahox gene *Cdx* is undetectable in all Euneoophora. Spiral cleavage has been lost in Acentrosomata, and the three centrosome-associated genes shown are undetectable in this group.

Our tree supports the now canonical view of Platyhelminthes as members of Lophotrochozoa, which was first shown using 18S rDNA data [6] and has subsequently received strong support from multigene phylogenies (e.g., [7]). Of perhaps greater interest is the finding of a strongly supported sister group relationship between the two species representing the order Catenulida and the remaining Platyhelminthes: the Rhabditophora (Figures 1, 2, 3, and 4). Rhabditophora share the convincing molecular synapomorphy of two changes in mitochondrial genetic code [8], and we provide phylogenomic confirmation of the monophyly of Platyhelminthes (Catenulida+Rhabditophora). Surprisingly, a convincing phenotypic synapomorphy of Platyhelminthes is still lacking [9, 10]. We have not considered the xenacoelomorphs, originally part of Platyhelminthes, as they have been shown by various means not to be part of the protostomes [11].

### Support for Platyzoa May Derive from a Long-Branch Attraction Artifact

While our ML tree supports Platyzoa ((Platyhelminthes, Gnathifera)(Gastrotricha)) [12–14] (Figure S1), with the rotiferans representing the larger group of Gnathifera, our Bayesian analyses, in common with two recent well-sampled phylogenomic studies of lophotrochozoan relationships [7, 11], show largely consistent support for Gastrotricha and Platyhelminthes being grouped together with Nemertea, Annelida, and Mollusca; Rotifera are outside of this clade (Figure 1). In our CAT+GTR+G4 analysis, Nemertea are sister group of Platyhelminthes+Gastrotricha. The exception to this finding is in our jackknife analysis, where the position of gastrotrichs relative to platyhelminths and other lophotrochozoan phyla is unresolved (Figure 2). In the signal dissection experiment, the fastest-evolving quartile of the data (most susceptible to long-branch attraction [LBA] or LBA artifact) supports Platyzoa (Figure 3, Q4), and this gives credence to the view of Platyzoa as arising from such a systematic error. Adopting measures to counter this problem with selected slowly evolving genes and well-fitted models (CAT+GTR+G4) rejects Platyzoa (Figure 3, Q1 and Q2).

### A Biflagellate Sperm Unites All Rhabditophora except Macrostomorpha

To date, the identity of the basalmost branching group of Rhabditophora has not been settled, with Macrostomorpha and Polycladida vying for this position [2, 7, 14–16]. Members of both of these groups possess the likely primitive character of spiral cleavage (absent in many more derived groups, see Figure 4) and also have entolecithal eggs, again a likely primitive character. Polycladida have a larval stage (present in both major clades of polyclads) that some consider homologous to the trochophore seen in several other lophotrochozoan phyla [17]. Macrostomorpha have aflagellate sperm; this contrasts with the remaining Rhabditophora, including Polycladida, which typically have a biflagellate sperm with a 9 × 2 + “1” pattern of microtubules and on this basis have been grouped as Trepaxonemata [1, 18]. Our data strongly support Macrostomorpha as sister group of all other rhabditophoran orders. Macrostomorpha are excluded from the monophyletic Trepaxonemata with posterior probability of 1.0 (Figure 1), jackknife support of 1.0 (Figure 3), and PhyML “SH-like” support [4] of 1.0 (Figure S1) as well as being found with pp = 1.0 in all four quartiles of the signal dissection experiment (Figure 3).

### Independent Evolution of Ectolecithal Eggs in Lecithoepitheliata and Euneoophora

Apart from Macrostomorpha and Polycladida, all rhabditophoran groups, including Lecithoepitheliata, are distinguished by ectolecithal eggs (yolk not incorporated into the embryonic blastomeres) and the associated characteristic (absent in Lecithoepitheliata) of an ovary structured into separate germary and vitellary areas. This assemblage of Rhabditophora with ectolecithal eggs is generally considered to constitute a clade called Neoophora [1, 19]. Lecithoepitheliata have been reconstructed as sister group of other Neoophora based on morphological characters [1] and limited marker molecular data [2, 20]. Lecithoepitheliata are split into freshwater-dwelling Prorhynchida and marine Gnosonesimida and may in fact be para- or polyphyletic [1, 2, 21]. In the only molecular study involving members of both taxa, they are presented as being grouped with other ectolecithal Platyhelminthes (i.e., members of the Neoophora), but Prorhynchida were found to be sister group of all other Neoophora, and Gnosonesimida as sister group of all other Neoophora except Prorhynchida [2]. This topology led these authors to support the monophyly of Neoophora and the single origin of ectolecithality.

Our study includes two members of Prorhynchida and, in striking contrast to most previous studies, places them not in Neoophora but as sister group of Polycladida, in accordance with [16]. The monophyly of Polycladida and Lecithoepitheliata/Prorhynchida is given maximum support in all analyses (Figures 1, 2, 3, S1, and S2).

This result has important implications for our understanding of the evolution of ectolecithality within flatworms: eggs with extraembryonic yolk cells would have evolved at least twice independently, once in Lecithoepitheliata (at least Prorhynchida) and once in the common ancestor of the remaining Neoophora. Neoophora excluding Lecithoepitheliata were named Euneoophora [2]. In further support of the monophyly of Euneoophora, we found that the parahox gene *Caudal*/*Cdx* was detectable in the transcriptomes of the lophotrochozoan phyla we sampled and also in the different orders of archoophorans, i.e., catenulids, macrostomorphans, polyclads, and lecithoepitheliates, but was undetectable in the transcriptomes of all included euneoophorans (Figure 4).

### Rhabdocoela, Not Proseriata, Are Likely to Be the Basalmost Euneoophoran Clade

The least confidently resolved part of the flatworm portion of our tree involves the relative positions of proseriates, rhabdocoels, and the remaining euneoophorans. In our CAT+GTR+G4 phylogeny of our complete dataset, Rhabdocoela are sister group of all other Euneoophora (pp = 1.0) (Figure 1), but with low jackknife support of 0.6 (Figure 2). Other analyses (ML) instead support Proseriata in this position (Figures S1 and S2), and this is in common with most previous analyses involving one or a few genes [2, 16, 20, 22–25].

On balance, we suggest that the basal Rhabdocoela solution is the most likely for two reasons. The first reason is the support it receives from the typically better-performing CAT+GTR+G4 model analysis over the PhyML analysis. The site-heterogeneous CAT model has been repeatedly shown to fit real data better than simpler models such as the site-homogenous model used in the ML analyses, and to be better able to overcome systematic error [11]. The second reason is that we observed stronger support for basal Rhabdocoela when analyzing the slowly evolving genes (Q1 and Q2); the more rapidly evolving genes support an association of Rhabdocoela and Tricladida/Bothrioplanida/Neodermata (Q3) or Rhabdocoela and Tricladida/Neodermata (Q4) (Figure 3). The support that this particular grouping receives in the analyses of more rapidly evolving genes seems likely to be due to an LBA artifact that leads to an incorrect association between the rhabdocoels and neodermatans, both of which have long branches. LBA is exacerbated by rapidly evolving genes [26]. In the fastest quartile of data (Q4; a priori most susceptible to LBA) the long-branched rhabdocoels move even closer to the long-branched Tricladida/Neodermata than the short-branched *Bothrioplana* (Figure 3).

### Loss of Centrosomes Defines a Group Including Planarians and Parasites

More reliably resolved is the position of Tricladida, which has strong support for a position closer to Neodermata and Bothrioplanida than either Rhabdocoela or Proseriata. None of the triclads, bothrioplanids, or neodermatans show any sign of spiral cleavage in their early embryogenesis, and the loss of this trait is a persuasive morphological character uniting this group (Figure 4). Recent studies have noted that genes including *SPD-2/Cep192*, *Nek2*, and *CCCAP*, which have an evolutionarily conserved role in centrosome formation across Metazoa, were missing from the planarian *Schmidtea mediterranea* as well as from the neodermatan *Schistosoma mansoni* yet were present in the macrostomorphan *Macrostomum lignano* [27]. This gene loss correlates with the loss of the centrosome in *Schmidtea* and possibly also in *Schistosoma*, and it was suggested that this loss of centrosomal genes is also implicated in the loss of the highly regulated spiral cleavage [27]. Thanks to our taxonomically broad sample of transcriptomes, we have been able to extend this analysis and show that three genes associated with centrioles, *SPD-2/Cep192*, *Nek2*, and *CCCAP*, are at least partly present in most of the more basally branching platyhelminth taxa for which we have transcriptomes but are undetectable in any of the Tricladida, Bothrioplanida, or Neodermata (Figure 4). The evidence for absence of a gene based on inevitably partial transcriptomes must not be overinterpreted, however, and we note that none of these three genes are found in the transcriptomes that we have produced for two lecithoepitheliates (Figure 4), which show a rather conserved spiral cleavage pattern [19].

### Identifying the Free-Living Ancestor of the Parasitic Neodermata

The monophyly of Neodermata with well-characterized apomorphies such as a secondary unciliated syncytial epidermis is undisputed [1]. It has long been clear that “Turbellaria” is a paraphyletic group and that the wholly parasitic Neodermata emerged from among free-living forms [1]. That said, the identity of the closest free-living relative of Neodermata has proven elusive. In early morphological phylogenies, Rhabdocoela (or members of Rhabdocoela) were considered to be sister group of Neodermata [1, 9, 28]. This relationship was not supported in subsequent molecular phylogenies using one or a few genes, in which a bewildering selection of higher flatworm taxa, e.g., Fecampiida, Prolecithophora, and Tricladida [29] or Rhabdocoela, Fecampiida, Prolecithophora, and Tricladida [16, 20, 23–25], were proposed as sister group of Neodermata. In a recent study using four genes and many taxa [2], and now in our own study using 1,347 genes, Bothrioplanida, previously considered close to or part of the Proseriata [1], are shown to be sister group of Neodermata (Figure 1).

### Relationships among the Neodermatan Groups

Neodermata comprise Monogenea, Cestoda, and Trematoda [1]. The interrelationships of these taxa has been debated, with Cestoda being considered sister group of either Monogenea ( = Cercomeromorpha) or Trematoda. The Cercomeromorpha hypothesis was rejected by phylogenetic analyses using 18S and 28S sequences [16, 25], and the alternative sister group relationship between Trematoda and Cestoda was supported by studies employing whole mitochondrial gene phylogenies [30, 31], by a microRNA study [32], by a multigene phylogeny using 312 gene models [33], and now by our own study. Surprisingly, a recent phylogenetic study using four genes and a large number of flatworm species supports the Cercomeromorpha hypothesis [2].

### Old and New Systematic Names

With the sister group relationship between Polycladida and Lecithoepitheliata/Prorhynchida demonstrated by our phylogenetic analysis (Figure 1), the taxon Neoophora, defined as encompassing all flatworms with ectolecithal eggs [1], has become polyphyletic and should therefore be noted with quotation marks, “Neoophora.” “Neoophora,” excluding Prorhynchida, are monophyletic in our analyses, and this clade has previously been named Euneoophora, characterized by the presence of ectolecithal eggs and by germaria and vitellaria as spatially separated organs [2].

We propose the name Amplimatricata *new taxon* for Polycladida+Lecithoepitheliata, based on the tendency in both groups for possession of an ample extracellular matrix [34]. Taking into account the remaining uncertainty over the monophyly of Lecithoepitheliata [2], Amplimatricata encompasses at least Polycladida+Prorhynchida. Acentrosomata *new taxon* is a clade consisting of Tricladida and its closely related taxa Prolecithophora and Fecampiida (all three taxa making up Adiaphanida [35]), Bothrioplanida, and Neodermata (Figure 4). The name is based on the implied absence of centrosomes in all of these taxa (Figure 4). Lacking strong similarities to serve as a clade-defining synapomorphy between Bothrioplanida and Neodermata, we use the name Bothrioneodermata *new taxon* to identify this monophyletic group (Figure 4).

### Conclusions

We have presented new transcriptomic data from 22 new species and produced a large and taxonomically complete dataset for assessing the relationships of Platyhelminthes. The majority of our conclusions are robust and are supported by different methods of analysis, high Bayesian posterior probabilities, and high jackknife support. The two instances of lower support concern the position of Platyhelminthes relative to other lophotrochozoan phyla and the early-branching position of rhabdocoels relative to other Euneoophora. The evidence against Platyzoa and support for early-branching Rhabdocoela by site-heterogeneous analyses (Figure 1) and by the slowest-evolving quartiles of the total dataset (Figure 3, Q1 and Q2) suggest that the alternatives, which are supported by the less-well-fitting site-homogenous analyses (Figures S1 and S2) and the faster-evolving quartiles of the data (Figure 3, Q3 and Q4), are the result of LBA. The suggested monophyly of Proseriata+Acentrosomata (Figure 4) might be tested further by the addition of the two additional members of Adiaphanida, Fecampiida and Prolecithophora, as well as the second clade of Lecithoepitheliata, Gnosonesimida, as there has been evidence that Lecithoepitheliata may be paraphyletic [1, 2].

## Author Contributions

B.E., C.B.C., C.N., E.B., F.L., K.A.R., J.G., M.A.T., and M.G. collected samples for RNA extraction. B.E., F.L., J.G., and K.A.R. extracted RNA. B.E. and M.J.T. assembled transcriptomes. B.T., C.D., N.S., and S.M. produced the OMA analyses. M.J.T. assembled the sets of orthologs and conducted phylogenetic analyses. B.E. and M.J.T. wrote the manuscript and designed the figures. All authors read and approved the final manuscript.

## Accession Numbers

The NCBI Sequence Read Archive experiment accession numbers for the data reported in this paper are SRX871300, SRX871445, SRX872404, SRX871533, SRX872402, SRX872327, SRX872365, SRX871508, SRX872321, SRX875739, SRX875742, SRX872403, SRX872314, SRX883021, SRX872398, SRX872347, SRX872356, SRX872362, SRX872414, SRX872416, SRX879690, SRX872410, SRX874324, and SRX875881.

## Supplemental Information

Supplemental Information includes three figures, one table, and Supplemental Experimental Procedures and can be found with this article online at http://dx.doi.org/10.1016/j.cub.2015.03.034.

## Supplemental Information

Document S1. Figures S1–S3 and Supplemental Experimental Procedures

Table S1. Species Names, Taxonomic Classification, Accession Numbers or Web Addresses, and Completeness Statistics All sequence data used in this study are listed with taxonomic classifications, accession numbers or download addresses, and completeness statistics. They are grouped in blocks designating whether the datasets were newly made for this study or were downloaded, and for which parts of the analyses they were used.

Document S2. Article plus Supplemental Information

## References

[1] Ehlers U. (1985). Das Phylogenetische System der Plathelminthes.

[2] Laumer C.E., Giribet G. (2014). Inclusive taxon sampling suggests a single, stepwise origin of ectolecithality in Platyhelminthes. Biol. J. Linn. Soc. Lond..

[3] Lartillot N., Lepage T., Blanquart S. (2009). PhyloBayes 3: a Bayesian software package for phylogenetic reconstruction and molecular dating. Bioinformatics.

[4] Guindon S., Dufayard J.F., Lefort V., Anisimova M., Hordijk W., Gascuel O. (2010). New algorithms and methods to estimate maximum-likelihood phylogenies: assessing the performance of PhyML 3.0. Syst. Biol..

[5] Stamatakis A. (2014). RAxML version 8: a tool for phylogenetic analysis and post-analysis of large phylogenies. Bioinformatics.

[6] Aguinaldo A.M.A., Turbeville J.M., Linford L.S., Rivera M.C., Garey J.R., Raff R.A., Lake J.A. (1997). Evidence for a clade of nematodes, arthropods and other moulting animals. Nature.

[7] Struck T.H., Wey-Fabrizius A.R., Golombek A., Hering L., Weigert A., Bleidorn C., Klebow S., Iakovenko N., Hausdorf B., Petersen M. (2014). Platyzoan paraphyly based on phylogenomic data supports a noncoelomate ancestry of spiralia. Mol. Biol. Evol..

[8] Telford M.J., Herniou E.A., Russell R.B., Littlewood D.T.J. (2000). Changes in mitochondrial genetic codes as phylogenetic characters: two examples from the flatworms. Proc. Natl. Acad. Sci. USA.

[9] Smith J.P.S., Tyler S., Rieger R.M. (1986). Is the Turbellaria polyphyletic?. Hydrobiologia.

[10] Egger B., Steinke D., Tarui H., De Mulder K., Arendt D., Borgonie G., Funayama N., Gschwentner R., Hartenstein V., Hobmayer B. (2009). To be or not to be a flatworm: the acoel controversy. PLoS ONE.

[11] Philippe H., Brinkmann H., Copley R.R., Moroz L.L., Nakano H., Poustka A.J., Wallberg A., Peterson K.J., Telford M.J. (2011). Acoelomorph flatworms are deuterostomes related to *Xenoturbella*. Nature.

[12] Cavalier-Smith T. (1998). A revised six-kingdom system of life. Biol. Rev. Camb. Philos. Soc..

[13] Hejnol A., Obst M., Stamatakis A., Ott M., Rouse G.W., Edgecombe G.D., Martinez P., Baguñà J., Bailly X., Jondelius U. (2009). Assessing the root of bilaterian animals with scalable phylogenomic methods. Proc. Biol. Sci..

[14] Wey-Fabrizius A.R., Herlyn H., Rieger B., Rosenkranz D., Witek A., Welch D.B.M., Ebersberger I., Hankeln T. (2014). Transcriptome data reveal syndermatan relationships and suggest the evolution of endoparasitism in Acanthocephala via an epizoic stage. PLoS ONE.

[15] Mallatt J., Craig C.W., Yoder M.J. (2010). Nearly complete rRNA genes assembled from across the metazoan animals: effects of more taxa, a structure-based alignment, and paired-sites evolutionary models on phylogeny reconstruction. Mol. Phylogenet. Evol..

[16] Mallatt J., Craig C.W., Yoder M.J. (2012). Nearly complete rRNA genes from 371 Animalia: updated structure-based alignment and detailed phylogenetic analysis. Mol. Phylogenet. Evol..

[17] Nielsen C. (2012). Animal Evolution.

[18] Justine J.-L., Littlewood D.T.J., Bray R.A. (2001). Spermatozoa as phylogenetic characters for the Platyhelminthes. Interrelations of the Platyhelminthes (The Systematics Association Special Volume Series 60).

[19] Martín-Durán J.M., Egger B. (2012). Developmental diversity in free-living flatworms. Evodevo.

[20] Joffe B.I., Kornakova E.E., Littlewood D.T.J., Bray R.A. (2001). Flatworm phylogeneticist: Between molecular hammer and morphological anvil. Interrelations of the Platyhelminthes (The Systematics Association Special Volume Series 60).

[21] Karling T.G. (1968). On the genus *Gnosonesima* Reisinger (Turbellaria). Sarsia.

[22] Littlewood D.T.J., Rohde K., Clough K.A. (1999). The interrelationships of all major groups of Platyhelminthes: phylogenetic evidence from morphology and molecules. Biol. J. Linn. Soc. Lond..

[23] Baguñà J., Carranza S., Paps J., Ruiz-Trillo I., Riutort M., Littlewood D.T.J., Bray R.A. (2001). Molecular taxonomy and phylogeny of the Tricladida. Interrelations of the Platyhelminthes (The Systematics Association Special Volume Series 60).

[24] Larsson K., Jondelius U. (2008). Phylogeny of Catenulida and support for Platyhelminthes. Org. Divers. Evol..

[25] Lockyer A.E., Olson P.D., Littlewood D.T.J. (2003). Utility of complete large and small subunit rRNA genes in resolving the phylogeny of the Neodermata (Platyhelminthes): implications and a review of the cercomer theory. Biol. J. Linn. Soc. Lond..

[26] Felsenstein J. (1978). Cases in which parsimony or compatibility methods will be positively misleading. Syst. Zool..

[27] Azimzadeh J., Wong M.L., Downhour D.M., Sánchez Alvarado A., Marshall W.F. (2012). Centrosome loss in the evolution of planarians. Science.

[28] Karling T.G., Riser N.W., Morse M.P. (1974). On the anatomy and affinities of the turbellarian orders. Biology of the Turbellaria.

[29] Willems W.R., Wallberg A., Jondelius U., Littlewood D.T.J., Backeljau T., Schockaert E.R., Artois T.J. (2006). Filling a gap in the phylogeny of flatworms: relationships within the Rhabdocoela (Platyhelminthes), inferred from 18S ribosomal DNA sequences. Zool. Scr..

[30] Park J.-K., Kim K.-H., Kang S., Kim W., Eom K.S., Littlewood D.T.J. (2007). A common origin of complex life cycles in parasitic flatworms: evidence from the complete mitochondrial genome of *Microcotyle sebastis* (Monogenea: Platyhelminthes). BMC Evol. Biol..

[31] Perkins E.M., Donnellan S.C., Bertozzi T., Whittington I.D. (2010). Closing the mitochondrial circle on paraphyly of the Monogenea (Platyhelminthes) infers evolution in the diet of parasitic flatworms. Int. J. Parasitol..

[32] Fromm B., Worren M.M., Hahn C., Hovig E., Bachmann L. (2013). Substantial loss of conserved and gain of novel MicroRNA families in flatworms. Mol. Biol. Evol..

[33] Hahn C., Fromm B., Bachmann L. (2014). Comparative genomics of flatworms (Platyhelminthes) reveals shared genomic features of ecto- and endoparastic Neodermata. Genome Biol. Evol..

[34] Rieger R.M., Tyler S., Smith J.P.S., Rieger G.E., Harrison F.W., Bogitsh B.J. (1991). Platyhelminthes: Turbellaria. Microscopic Anatomy of Invertebrates, Volume 3: Platyhelminthes and Nemertinea.

[35] Noren M., Jondelius U. (2002). The phylogenetic position of the Prolecithophora (Rhabditophora, ‘Platyhelminthes’). Zool. Scr..

