# Supplementary material for: A Transcriptomic-Phylogenomic Analysis of the Evolutionary Relationships of Flatworms
Source: Curr Biol. 2015 May 18;25(10):1347–53. doi: 10.1016/j.cub.2015.03.034 (PMC4446793; doi:10.1016/j.cub.2015.03.034)
Supplement: Document S1. Figures S1–S3 and Supplemental Experimental Procedures [file mmc1.pdf]

Current Biology
Supplemental Information

A Transcriptomic-Phylogenomic Analysis of the Evolutionary Relationships of Flatworms

Bernhard Egger, François Lapraz, Bartłomiej Tomiczek, Steven Müller, Christophe Dessimoz, Johannes Girstmair, Nives Škunca, Kate A. Rawlinson, Christopher B. Cameron, Elena Beli, M. Antonio Todaro, Mehrez Gammoudi, Carolina Noreña, and Maximilian J. Telford

Supplemental Information

Figure S1: Phylogeny produced using PhyML with the site-homogenous LG+G4 model on full 107,659 amino acids alignment. Values at nodes indicate SH-like support [4]. Major differences compared to Fig. 1 are the clade of Platyhelminthes, Gastrotricha and Rotifera (Platyzoa) and the reversed positions of Rhabdocoela and Proseriata. This may be due to

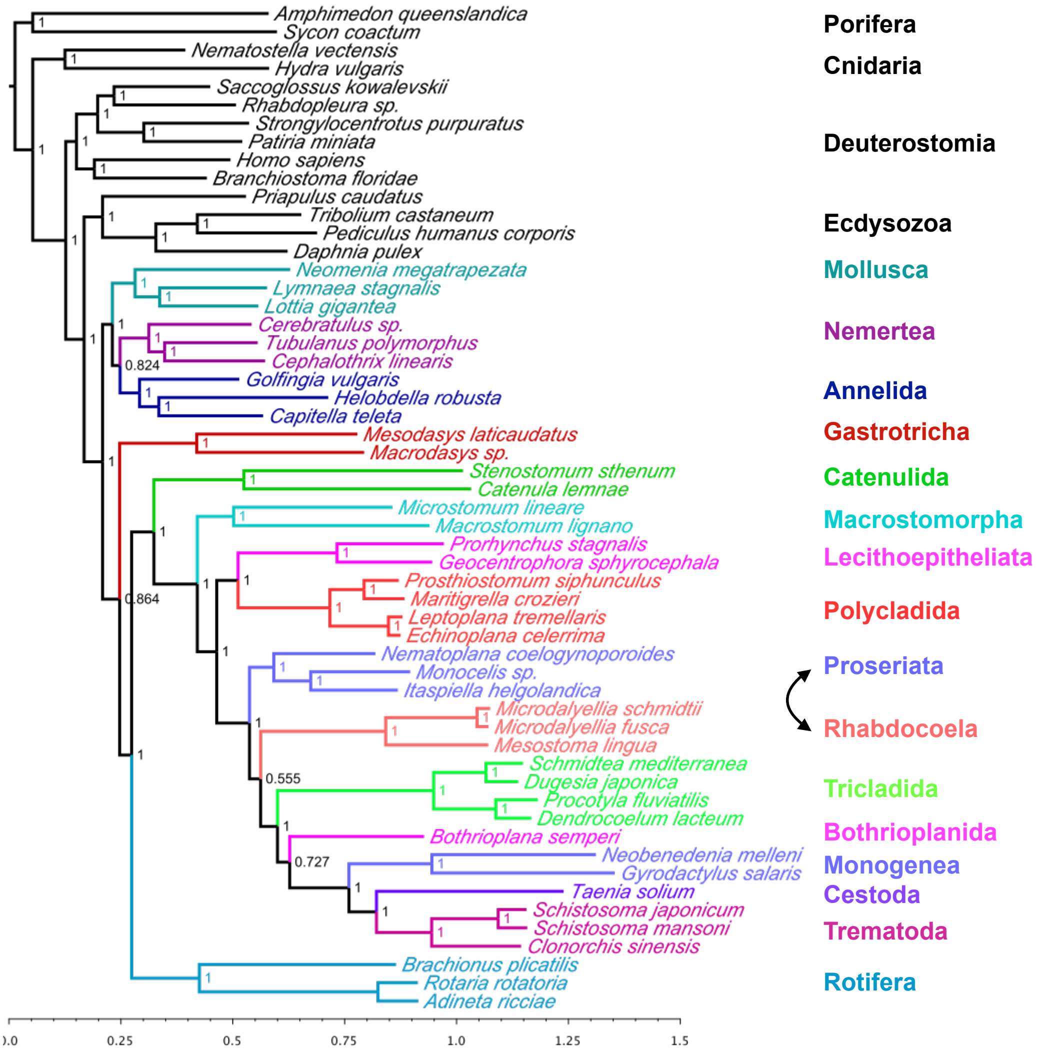

Long Branch Attraction between groups of 'platyzoans' and between Rhabdocoela and Neodermata. LBA has been shown to be more prevalent with the site-homogenous model used here. Scale bar indicates number of substitutions per site.

Figure S2: Phylogeny produced using RAxML with the site-homogenous LG+G4 model on full 107,659 amino acids alignment [5]. Major differences compared to Fig. 1 are the clade of Platyhelminthes, Gastrotricha and Rotifera (Platyzoa) and the reversed positions of Rhabdocoela and Proseriata. This may be due to Long Branch Attraction between groups of 'platyzoans' and between Rhabdocoela and Neodermata. LBA has been shown to be more prevalent with the site-homogenous model used here. Scale bar indicates number of substitutions per site.

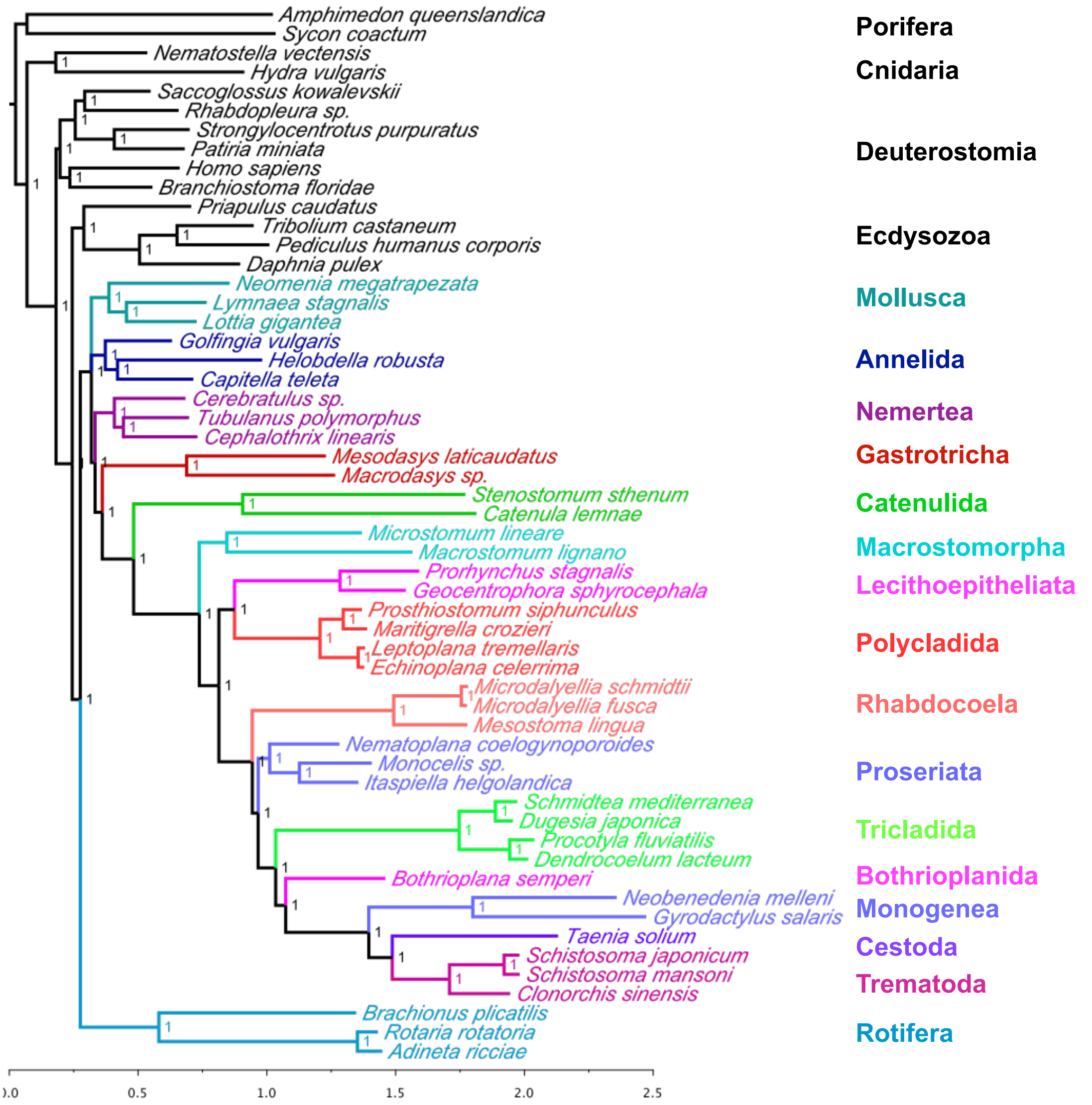

Figure S3: Graph showing the relative rates of substitution of all 1348 genes ordered by rate as used in the phylogenetic signal dissection experiment. The division of genes into four quartiles is indicated. The number of genes making up the faster evolving quartiles is larger because the final datasets were produced after deleting sites with more than a set percentage of missing data. The faster evolving genes had more missing data (or were less easy to align into regions conserved across all taxa). The final 4 datasets all contained the same number of positions.

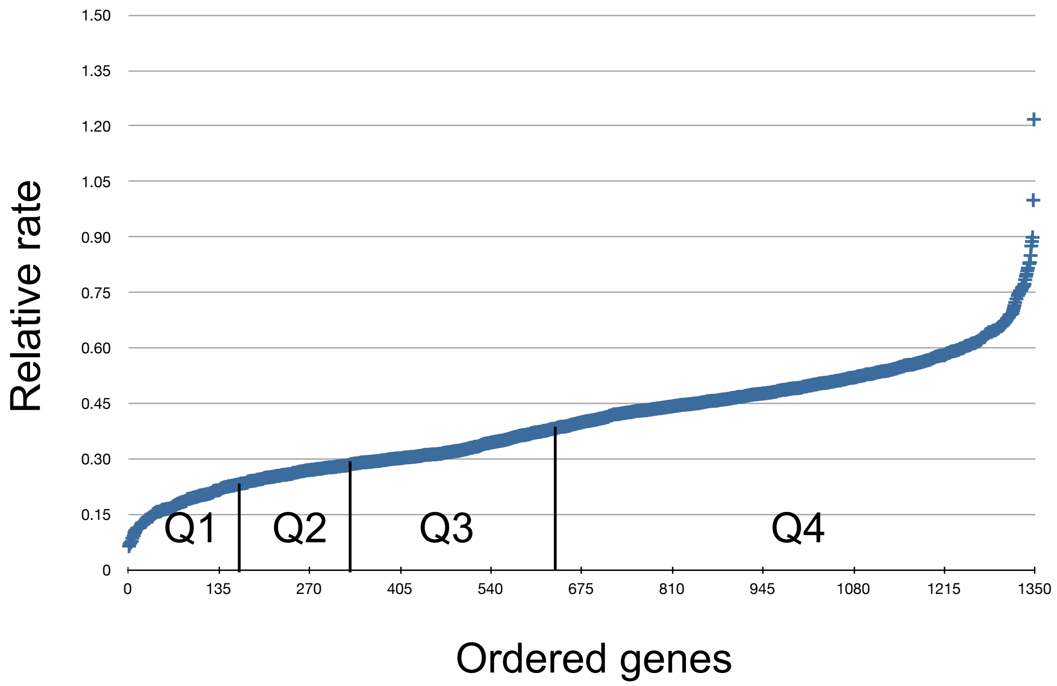

Supplemental Experimental procedures

Specimen collection and determination

Polyclad flatworms were collected from their substrate with a soft brush and transferred into a water-filled container [S1]. For marine interstitial flatworms (*Itaspiella helgolandica*, *Monocelis* sp., *Nematoplana coelogynoporoides*) sand samples were collected into lockable plasticware containers and later extracted in the laboratory with a 1:1 solution of $MgCl_2*6H_2O$ and sea water and filtered through 40-100 µm meshes [S2]. The content of the meshes was flushed with seawater into petri dishes and animals of interest were sorted into embryo dishes under a binocular microscope. Freshwater samples – containing water plants, mud or sand – were poured into petri dishes, further diluted with water if necessary and animals of interest were searched for under a binocular microscope.

Extraction of *Mesodasys laticaudatus* (Macrodasyida, Cephalodasyidae) was done by the narcotization-decantation technique using a 7% magnesium chloride solution. Animals were allowed to recover for 2 hours in sea water.

Species determination was carried out with live animals using either whole animals under a binocular or squeeze preparations under a compound microscope [S3]. Literature used for species determination were taxonomic guides [S4-S7], monographs on particular groups [S8-S10] and specific taxonomic accounts of considered species [S11-S12], all facilitated by the excellent Turbellarian Database (http://turbellaria.umaine.edu/).

RNA extraction and sequencing

For one sample (*Prorhynchus stagnalis*, BioProject PRJNA275072) kept in RNAlater (Life Technologies), a Nucleospin RNA XS kit (Macherey-Nagel, Düren, Germany) was used. For all other samples a TRIzol Reagent (Life Technologies, Carlsbad, CA)/TRI Reagent (Sigma-Aldrich, St. Louis, MO) based RNA extraction protocol was used on live animals or dissected tissues, following manufacturers' protocols. Total RNA was stored at -80 °C until sent for sequencing (The Centre for Applied Genomics: The Hospital for Sick Children, Toronto, Canada), where cDNA libraries were prepared using Illumina TruSeq kits (Illumina Inc., San Diego, CA). For three samples with low amounts of RNA (*Catenula lemnae*, *Geocentrophora sphyrocephala* and the second *Prorhynchus stagnalis* sample, BioProject PRJNA275317), SMART mRNA amplification kits (Clontech Laboratories Inc., Mountain View, CA) followed by Nextera XT kits (Illumina) were used. The libraries were sequenced on an Illumina HiSeq 2000/2500 producing 100 bp paired end reads. In total, 18 flatworms, a nemertean, a sipunculid, a gastrotrich, a priapulid and a pterobranch were newly sequenced for this study, for all accession numbers see Table S1.

Transcriptome assembly and peptide prediction

After quality assessment with FastQC (http://www.bioinformatics.babraham.ac.uk/projects/fastqc/) it was determined using PRINSEQ lite [S13] that the first 12 nucleotides needed to be trimmed off the 100 bp reads. Assembly of the trimmed paired reads was done using Trinity v20130225 [S14] using the flag '--min_kmer_cov 2' in addition to default parameters. To test for the presence of cross contamination between libraries run on the same flow cell, we used the bowtie software (http://bowtie-bio.sourceforge.net) and a custom script to identify any assembled transcript with fewer than four read matches which were discarded. In addition we discarded all transcripts in which the number of reads from the intended species matching the transcript was not at least 5 times greater than the number of matches to the transcript from reads from any of the other potentially contaminating species.

For peptide predictions for all nucleotide data sets (i.e. including those publically available), the Trinity script 'transcripts_to_best_scoring_ORFs.pl' was run on the nucleotide data set, keeping all ORFs >100aa. For all peptide datasets cd-hit [S15] was used to reduce redundancy by clustering sequences with a global sequence identity of >95%. All subsequent analyses, including the phylogenetic analyses were based on amino acid sequences.

<u>OMA analysis of homologous genes.</u>

In brief,

i) We processed the proteomes from 52 species using the OMA software (http://omabrowser.org) to identify 3,164 sets of orthologous proteins with at least 28 representative sequences.

ii) for each of these 3,164 sets of orthologs we identified additional orthologs from 35 new species to give 87 species in total.

iii) we selected 55 species from the total of 87 species, eliminating taxa with lots of missing data.

iv) we produced one tree per set of orthologs and kept only gene sets with large sets of monophyletic platyhelminths: 1,348 sets of orthologs.

Non-redundant peptide datasets from 52 species including 27 species of platyhelminths, 8 species of non-platyhelminth lophotrochozoans, and 17 other ecdysozoan, deuterostome, diploblast and sponge species as outgroups (see Table S1) were processed by the OMA software using default settings to identify sets of Orthology Groups (= OG; sets of genes in which all representatives are orthologous to all other members). The all-against-all comparisons of sequences were run in parallel on the UCL Computer Science cluster. Using OMA we were able to identify 230,759 OGs. From these we selected 3,164 OGs with a minimum of 28 species represented (>50% of species with a member of the OG).

<u>Assembling larger sets of orthologs for phylogenetic analyses</u>

As we were able to add newly available data from our own sequenced transcriptomes as well as recently available public data we devised a pipeline for adding new sequences to our existing orthology groups. Running the OMA all-against-all is extremely computationally

intensive and the time taken increases quadratically with respect to the number of species, we decided, therefore, to follow a considerably quicker approach that focussed only on genes that match the 3,164 previously identified OGs.

Using custom perl scripts, for each existing OG we aligned the OG sequences (OG-ALIG) and from this alignment produced a Hidden Markov Model (OG-HMM) using Hmmer1.3b1 [S16]. We next searched the set of sequences from the initial OG with its own OG-HMM to find the score of the lowest sequence match, this lowest score then provided an OG specific cutoff for searching for additional sequences from other sets of peptides.

For each species, an HMM search using each OG-HMM was conducted and the top 3 sequences with a match greater than the OG specific cutoff were kept. Next, for each OG, the standalone version of OMA was run on the collection of potential hits from all new species plus the original constituents of the OG.

OMA standalone found the orthology groups present in each collection of sequences derived from the initial HMM search as well as additional OGs present thanks to the relatively low cutoff used. Sometimes, more than one of the new OGs produced in this way contained identical sequences because the low cutoff meant members of one orthology group could also be picked up by a second. To disentangle these, all instances of OGs in which any sequence also appears in another OG were merged and OMA standalone run again on the merged set of sequences. This approach allowed all paralogs to be disentangled into separate and unique OGs. The end result was 8,424 new OGs, the increase in total number of OGs is due to the presence of paralogs for some genes, typically each paralog had a small number of sequences when compared to the original OG it was based on.

At this stage we had data from 87 taxa with different levels of completeness. To improve the overall quality of the concatenated alignment the 32 least complete or redundant taxa were now deleted (e.g. we kept only the most complete species of the genus *Brachionus*) to leave 55 species. Some lower quality flatworms were retained due to their interest within the scope of the project (see Table S1). The OGs were now selected for further analysis only if they contained sequences from at least 25 species. 2,528 OGs were kept at this stage.

To reduce the likelihood of contaminating sequences or the presence of paralogs within the Rhabditophora, using custom PERL scripts, we cut any rhabditophoran sequences which did not cluster with the main clade of Rhabditophora on a tree constructed for each OG. Each tree was built based on a muscle alignment, trimmed with trimAl [S17] and analysed using MrBayes v 3.2.2 [S18] (settings: prset applyto = (all) aamodelpr = mixed; lset rates = gamma ngammacat = 5; nruns = 2 nchains = 2 ngen = 50000 samplefreq = 10 Diagnfreq = 1000 Burninfrac = 0.5 stoprule = yes Stopval = 0.1 Starttree = parsimony). The OG was only kept if there were more than 10 rhabditophoran sequences clustered in a monophyletic grouping on the tree and if this largest clade contained at least 4 times as many species as the next

largest rhabditophoran clade. All rhabditophoran sequences not members of this largest clade were deleted as potential contaminants/paralogs. After this cleaning procedure there remained 1,348 OGs. We did not include the Catenulida within this requirement in order to allow us to test the monophyly of the Platyhelminthes.

Using the alignment for each remaining OG, a maximum-likelihood tree was calculated using PhyML [4]. PhyML settings used were -o tlr (tlr: tree topology (t), branch length (l) and rate parameters (r) are optimised) -a e (alpha parameter of gamma distribution is estimated) -c 5 (5 gamma rate categories), the substitution model was LG. The total length of that tree (in estimated substitutions per position across all branches) was divided by the number of taxa on the tree to give an estimate of the rate of evolution for each gene. Genes were concatenated in order of their evolutionary rates (see section 'Phylogenetic signal dissection') to produce an overall alignment of 563,188 positions. This was processed to keep only those individual positions with a minimum of 60% occupancy producing a final alignment with 107,659 positions. The overall completeness of the species in the trimmed alignment ranged from 15%-97% with an average of 73% completeness (see Table S1).

<u>Phylogenetic tree reconstruction</u>

Trees were constructed using PhyloBayes 3.3e [3]. The site heterogeneous CAT+GTR+G4 mixture model was used. This model has repeatedly been shown using cross-validation to be optimal for large datasets such as that presented which has the capacity to provide estimates of the large number of parameters required [11,S19-S20].

Two independent runs were performed with a total length of >4000 cycles. To construct the tree, the first 500 cycles were discarded as burn-in, and the topology and posterior consensus support was computed on the remaining trees (Fig. 1).

Trees were also reconstructed using the maximum likelihood approach using PhyML v 3.0. The LG substitution model was selected, the proportion of invariable sites was estimated and a gamma distribution with 4 categories used. An approximate likelihood ratio test using SH-like supports [4] was conducted to provide estimates of support for clades on the best tree (Suppl. Fig. 1).

Trees were also reconstructed using the maximum likelihood approach using RAxML 8 [5]. The CAT GTR substitution model was selected. n.b. the RAXML 'CAT' has no relation to the phylobayes 'CAT' model, it is instead related to the gamma correction.

<u>Jackknifing</u>

In order to provide an alternative estimate of the support for the clades within the tree we used a jackknife approach. 100 jackknife samples were produced by sampling 20,000 positions at random from the full data set. Each data set was analysed for 300 cycles using

the CAT+GTR+G4 analysis of PhyloBayes and a consensus tree produced for each sample using a burn-in of 200 cycles. The 100 consensus trees produced in this way were collated and a master consensus was produced which represents the overall consensus jackknife tree (Fig. 2).

Phylogenetic signal dissection

To gauge the effects of using data sets with different evolutionary rates on the support for different clades in our tree we divided the total alignment, for which genes had been ordered based on rate of evolution from slowest to fastest, into four quartiles, Q1-Q4. Q1 contains the 25% of positions from the slowest evolving genes in the alignment, Q2 the next 25% etc. These 4 quartiles were each used to reconstruct a phylogenetic tree as previously described (Fig. 3).

Supplemental References

S1. Prudhoe, S. (1985). A Monograph on Polyclad Turbellaria. First Edition (Oxford: Oxford University Press).

S2. Pfannkuche, O., and Thiel, H. (1988). Sample processing. In Introduction to the study of meiofauna, R.P. Higgins, and H. Thiel, eds. (Washington: Smithsonian Institution), pp. 134-145.

S3. Westheide, W., and Purschke, G. (1988). Organism processing. In Introduction to the study of meiofauna, R.P. Higgins, and H. Thiel, eds. (Washington: Smithsonian Institution), pp. 146-160.

S4. Luther, A. (1960). Die Turbellarien Ostfennoskandiens I. Acoela, Catenulida, Macrostomida, Lecithoepitheliata, Prolecithophora, und Proseriata. Fauna Fenn. Hels. 7, 1-155.

S5. Young, J.O. (2001). Keys to the freshwater microturbellarians of Britain and Ireland with notes on their ecology. Freshw. Biol. Ass. Sci. Publ. 59, 1-142.

S6. Ax, P. (2008). Plathelminthes aus Brackgewässern der Nordhalbkugel. First Edition (Mainz: Franz Steiner Verlag).

S7. Streble, H., and Krauter, D. (2008). Das Leben im Wassertropfen. Eleventh Edition (Stuttgart: Franckh-Kosmos Verlag).

S8. Lang, A. (1884). Die Polycladen (Fauna und Flora des Golfes von Neapel). First Edition (Leipzig: W. Engelmann).

S9. Luther, A. (1955). Die Dalyelliiden (Turbellaria Neorhabdocoela). Acta Zool. Fenn. 87, 1-337.

S10. Faubel, A. (1983). The Polycladida, Turbellaria. Proposal and establishment of a new system. Part 1. The Acotylea. Mitt. Hamb. Zool. Mus. Inst. 80, 17-121.

S11. Nuttycombe, J.W. (1956). The Catenula of the Eastern United States. Amer. Midl. Nat. 55, 419-433.

S12. Borkott, H. (1970). Geschlechtliche Organisation, Fortpflanzungsverhalten und Ursachen der sexuellen Vermehrung von *Stenostomum sthenum* nov. spec. (Turbellaria,

Catenulida). Mit Beschreibung yon 3 neuen *Stenostomum*-Arten. Z. Morph. Tiere 67, 183-262.

S13. Schmieder, R., and Edwards, R. (2011). Quality control and preprocessing of metagenomic datasets. Bioinformatics 27, 863-864.

S14. Haas, B.J., Papanicolaou, A., Yassour, M., Grabherr, M., Blood, P.D., Bowden, J., Couger, M.B., Eccles, D., Li, B., Lieber, M., et al. (2013). De novo transcript sequence reconstruction from RNA-seq using the Trinity platform for reference generation and analysis. Nature Prot. 8, 1494-1512.

S15. Li, W., and Godzik, A. (2006). Cd-hit: a fast program for clustering and comparing large sets of protein or nucleotide sequences. Bioinformatics 22, 1658-1659.

S16. Finn, R.D., Clements, J., and Eddy, S.R. (2011). HMMER web server: interactive sequence similarity searching. Nucl. Acids Res. Web Server Issue 39, W29-W37.

S17. Capella-Gutiérrez, S., Silla-Martínez, J.M., and Gabaldón, T. (2009). trimAl: a tool for automated alignment trimming in large-scale phylogenetic analyses. Bioinformatics 25, 1972-1973.

S18. Ronquist, F., and Huelsenbeck J.P. (2003). MrBayes 3: Bayesian phylogenetic inference under mixed models. Bioinformatics 19, 1572-1574.

S19. Telford, M.J., and Copley, R.R. (2011). Improving animal phylogenies with genomic data. Trends Genet. 27, 186-195.

S20. Telford, M.J., Lowe, C.J., Cameron, C.B., Ortega-Martinez, O., Aronowicz, J., Oliveri, P., and Copley, R.R. (2014). Phylogenomic analysis of echinoderm class relationships supports Asterozoa. Proc. R. Soc. Lond. B Biol. Sci. 281, 20140479.
